# An activation domain of plasmid R1 TraI protein delineates stages of gene transfer initiation

**DOI:** 10.1111/j.1365-2958.2011.07872.x

**Published:** 2011-11-08

**Authors:** Silvia Lang, Paul C Kirchberger, Christian J Gruber, Adam Redzej, Sandra Raffl, Guenther Zellnig, Klaus Zangger, Ellen L Zechner

**Affiliations:** 1 University of Graz, Institute of Molecular BiosciencesHumboldtstrasse 50, 8010 Graz, Austria; 2 University of Graz, Institute of ChemistryHeinrichstrasse 28, 8010 Graz, Austria; 3 University of Graz, Institute of Plant SciencesSchubertstrasse 51, 8010 Graz, Austria

## Abstract

Bacterial conjugation is a form of type IV secretion that transports protein and DNA to recipient cells. Specific bacteriophage exploit the conjugative pili and cell envelope spanning protein machinery of these systems to invade bacterial cells. Infection by phage R17 requires F-like pili and coupling protein TraD, which gates the cytoplasmic entrance of the secretion channel. Here we investigate the role of TraD in R17 nucleoprotein uptake and find parallels to secretion mechanisms. The relaxosome of IncFII plasmid R1 is required. A ternary complex of plasmid *oriT*, TraD and a novel activation domain within the N-terminal 992 residues of TraI contributes a key mechanism involving relaxase-associated properties of TraI, protein interaction and the TraD ATPase. Helicase-associated activities of TraI are dispensable. These findings distinguish for the first time specific protein domains and complexes that process extracellular signals into distinct activation stages in the type IV initiation pathway. The study also provided insights into the evolutionary interplay of phage and the plasmids they exploit. Related plasmid F adapted to R17 independently of TraI. It follows that selection for phage resistance drives not only variation in TraA pilins but diversifies TraD and its binding partners in a plasmid-specific manner.

## Introduction

Bacterial type IV secretion systems (T4SS) have highly versatile functions due to their ability to transmit both proteins and nucleoprotein conjugates across the cell envelope. Type IV secretion has broad clinical significance not only for delivering bacterial toxins or effector proteins directly into targeted host cells, but also for direct involvement in phenomena such as biofilm formation and the rapid horizontal spread of antibiotic resistance genes among the microbial community (de la Cruz and Davies, 2000; Ghigo, 2001; McGowan, 2001; Backert and Meyer, 2006). Conjugation systems are the largest and most widely distributed of the T4 subfamilies. These systems are responsible for plasmid conjugation in Gram-negative and Gram-positive bacteria, as well as the transfer of integrated conjugative elements, which are phage-like sequences that have been integrated into the bacterial chromosome. The extensively studied T4S system of *Agrobacterium tumefaciens* is related to conjugation paradigms and used by this soil borne Gram-negative bacterium to genetically transform plants. Our current understanding of the mechanistic principles of T4 secretion is due to extensive research of these DNA delivery systems (Alvarez-Martinez and Christie, 2009).

The process involves three functional substructures: cell surface pili or adhesins that mediate contact between cells, a transport channel that conducts substrates across the bacterial cell envelope, and a type IV coupling protein (T4CP) that recruits secretion substrates to the cytoplasmic entrance of the secretion channel. The general mechanism of conjugative plasmid transfer is well-characterized. Multiple proteins assemble on the plasmid origin of transfer (*oriT*) to form the relaxosome (de la Cruz *et al*., 2010). This stable complex prepares the single-strand of plasmid DNA destined for transfer (T-strand) via the nicking-closing activity of a relaxase enzyme. Initiation of transfer requires cleavage of the phosphodiester bond at a specific position, *nic*, within *oriT.* The reaction is mediated by a tyrosine residue of the relaxase, so that a covalent tyrosinyl–DNA adduct is formed. This nucleoprotein complex is specifically recognized by the plasmid-encoded T4CP and actively pumped through the transport apparatus in a reaction requiring ATP. Once in the recipient, the relaxase–ssDNA intermediate restores the original circular plasmid molecule after termination of transfer via reversion of the strand transfer reaction. Finally, stabilization of the original plasmid DNA strands by conjugative replication occurs in both donor and recipient cells.

The process has enormous importance in human health care as a major vehicle of antibiotic resistance spread among pathogens and commensal bacteria alike (Baquero, 2004; Norman *et al*., 2009). Accordingly research has focused on gaining detailed knowledge of the initiation stage of T4S and its control. Recent advances provide details about the recruitment and recognition process of secretion substrates by T4CPs (Nagai *et al*., 2005; Schulein *et al*., 2005; Vergunst *et al*., 2005; Parker and Meyer, 2007; Lang *et al*., 2010). T4CPs mediate multiple protein–protein interactions with cytoplasmic and inner membrane components of the secretion system (Gomis-Rüth *et al*., 2001; Schröder and Lanka, 2003; Cascales and Christie, 2004; Alvarez-Martinez and Christie, 2009). Experiments designed to detect the mutual modulation of protein activities, stability and localization due to these interactions will be key to defining productive docking contacts between the relaxosome and the conjugative pore, and to reconstructing the initiation pathway. Correct progression of conjugative DNA processing by the relaxosome indeed requires regulatory interactions with the T4CP (Tato *et al*., 2007; Mihajlovic *et al*., 2009; Sut *et al*., 2009; Wong *et al*., 2011). Discovering the nature of the interactions which lead to channel opening and productive entry of macromolecular secretion substrates to the transport apparatus is much more challenging, largely because macromolecules are transferred in response to cell contact and environmental cues that remain poorly defined. To move forward we sought a whole cell activity assay that involved some or all of the proteins necessary for conjugation, but what may be subject to simpler regulation. Infection by a male-specific bacteriophage (Loeb, 1960) was a promising choice for F-like paradigms because the phage life cycle depends not only on conjugative pili but also on the T4CP (Valentine *et al*., 1969; Schoulaker and Engelberg-Kulka, 1978).

R17 phage adhere to F-like conjugative pili via the phage attachment (A) protein (Roberts and Steitz, 1967). In a subsequent reaction known as eclipse, the viral RNA genome dissociates from its coat protein and is transiently sensitive to RNase (Paranchych, 1975). Adsorption can occur on isolated pili but eclipse requires their cellular attachment (Valentine and Strand, 1965). A processed form of protein A, covalently linked to the 3′ end of the phage RNA, pilots the nucleoprotein complex from the capsid into the cell (Krahn *et al*., 1972; Wong and Paranchych, 1976). Entry may occur through retraction of pili, or via the pilus lumen following pilin rearrangements at the site of attachment (Marvin and Hohn, 1969; Paranchych *et al*., 1971). Penetration of multiple copies of this nucleoprotein complex to the host cytosol is followed by viral replication, packaging and cell lysis.

Early work identified several plasmid proteins essential for the phage life cycle including TraA pilin, the mating pair formation (Mpf) system involved in pilus biogenesis, lytic transglycosylase P19, and importantly, the T4CP TraD (Valentine *et al*., 1969; Schoulaker and Engelberg-Kulka, 1978; Bayer *et al*., 1995). It follows that the interaction of R17 phage with a piliated host conveys exogenous signals to the cell interior that activate the T4CP for nucleoprotein trafficking. In this study, we demonstrate that T4CP-dependent uptake of R17 RNA–protein A complexes by the plasmid R1 system involves docking interactions between the T4CP and its cognate relaxosome. Phage sensitivity was then used to explore whether activation of nucleoprotein import could be uncoupled from any or all DNA processing reactions necessary for nucleoprotein export. We find that host cells are vulnerable to infecting phage only through a T4S machinery that is also competent for conjugative DNA transfer. Finally, the T4CP and a partial complex of the relaxosome was found to have a key role in transmission of exogenous signals into activation of nucleoprotein transfer.

## Results

### Host cell sensitivity to bacteriophage R17 requires the R1-16 relaxosome

**Fig. 1:**
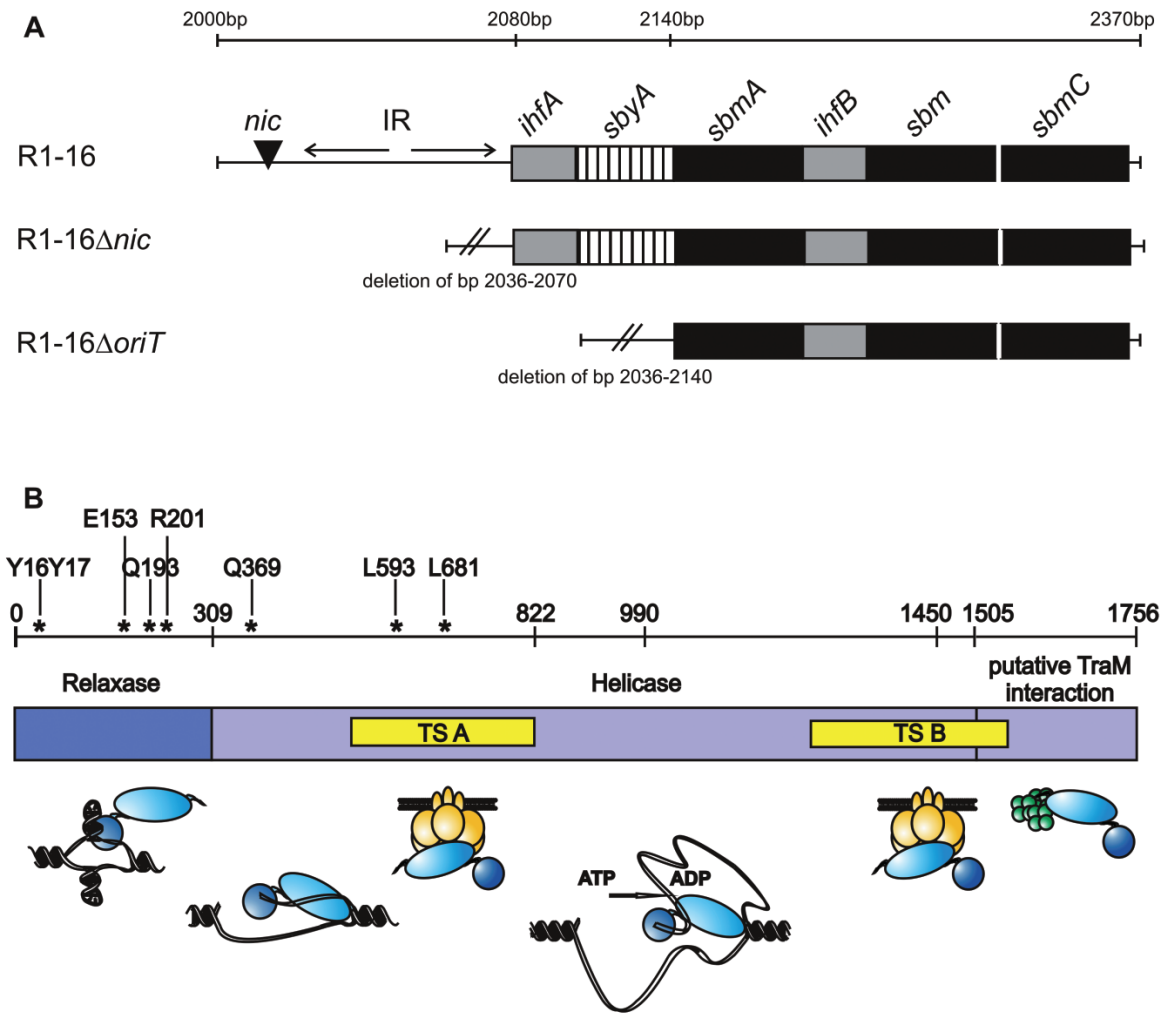
Schematic representation of the *oriT* deletion variants and the functional domains of TraI. A. DNA sequences important to TraI binding and strand cleavage include *nic* (black triangle), and the inverted repeat (IR, arrows). The binding sites for the accessory proteins IHF (*ihfA, ihfB*), TraY (*sbyA*) and TraM (*sbmA*, *sbmB*, *sbmC*) are illustrated. To create the deletions, *loxP-tetRA-loxP* cassettes replaced the sequences indicated [numbering according to Graus-Goldner *et al*. (1990)]. Subsequent expression of Cre recombinase *in trans* removed the cassettes from the R1-16 deletion derivatives. B. TraI domain N_1-309_ (*blue*) catalyses the relaxase reaction and contains the relaxase-associated ssDNA binding site. The helicase-associated ssDNA binding activity (N_1-822_) is independent of conserved helicase motifs (positions 990–1450). Negative cooperativity in ssDNA binding is observed between the relaxase and helicase-associated sites. Independently functional translocation signals TSA (positions 530–816) and TSB (1255–1564) mediate T4CP recognition (*yellow*). The C-terminal 252 residues may contain a putative interaction site for TraM (*green*). Positions of mutant variants used in this study are shown (*).

Early research of the R17 phage life cycle used *Escherichia coli* hosts carrying plasmid F, and in some cases the fertility derepressed variants of R1 (i.e. R1-16 or R1-19). Plasmid-specific differences in host phage sensitivity were described in numerous studies (Willetts and Maule, 1986). We chose to develop this model rigorously for R1 proteins, and tested whether host sensitivity required the R1-16 plasmid *oriT* and components of the relaxosome in addition to the T4CP TraD. In F-like systems the secreted protein TraI is a bifunctional relaxase that cleaves one plasmid strand and pilots the DNA to the recipient, and a helicase that is essential for transfer (de la Cruz *et al*., 2010). Additional relaxosome proteins that bind *oriT* with sequence specificity are the *E. coli* IHF and plasmid proteins TraM and TraY (Mihajlovic *et al*., 2009). Mutant derivatives of R1-16 lacking DNA sequences essential to assembly of a functional relaxosome were generated. We deleted 34 bp of *oriT* spanning the site of TraI relaxase-catalysed cleavage (R1-16Δ*nic*) (Fig. 1A). This eliminated *nic*, the inverted repeat (IR) and key bases for TraI recognition (Williams and Schildbach, 2006). A second construction removed 104 bp of *oriT* including *nic* and *ihfA* and *sby* binding sites for IHF and TraY (R1-16Δ*oriT*) (Fig. 1A). Single gene deletions in R1-16 eliminated the plasmid relaxosome protein components (Δ*traM*, Δ*traY* or Δ*traI*). A plaque assay for host cell R17 sensitivity (R17^S^) confirmed the requirement for the R1-16 T4CP TraD, as expected (Table 1). Mutation of the NTP-binding Walker A box in TraDK198T blocked complementation of the R17^S^ phenotype. The test screen of hosts carrying mutant R1-16 derivatives (Δ*nic*, Δ*oriT*, Δ*traM*, Δ*traY* or Δ*traI*) confirmed that relaxosome reconstitution is important for effective phage R17 infection (Table 1). The data argue most strongly for a role for TraI since R17^S^ of the R1-16Δ*traI* host was effectively complemented *in trans*. We also verified normal transfer (*tra*) gene expression from the R1-16Δ*nic* and R1-16Δ*oriT* mutant plasmids by measuring highly efficient conjugative mobilization of a coresident *oriT*_R1_ plasmid (not shown). Complementation of the conjugative self transfer of the R1-16Δ*traM* and R1-16Δ*traY* with wild-type expression *in trans* was 10^−4^ to 1 transconjugant per donor cell respectively, in good agreement with prior observation (Maneewannakul *et al*., 1996; Pölzleitner *et al*., 1997). Expression *in trans* failed to restore efficient R17^S^ to the host for these mutant derivatives. The transcriptionally repressed wild-type plasmid R1 normally transfers with a similar frequency of 10^−3^. Consistent with the mutant derivatives, plaque formation with R1-carrying hosts was below the level of detection. Results of this assay therefore cannot support or rule out a direct contribution of R1 TraM or TraY to host cell phage infection.

**Table 1:** R17 phage sensitivity of *E. coli*[R1-16] requires a functional relaxosome and NTP binding by T4CP TraD

Protein requirements				

Plasmid	Complementation^a^	Infection level^b^	Plaque morphology	pfu ml−1^c^
R1-16	−	+++	Clear	1.5 × 10^10^
R1-16Δ*traD*	−	−	None	n.d.
R1-16Δ*traD*	R1 TraD	++	Opaque	1.5 × 10^10^
R1-16Δ*traD*	R1 TraDK198T	−	None	n.d.
R1-16Δ*traM*	−	−	None	n.d.
R1-16Δ*traM*	R1 TraM	−	None	n.d.
R1-16 M13	−	−	None	n.d.
R1-16Δ*traY*	−	+	Very opaque	n.d.
R1-16Δ*traY*	R1 TraY	+	Very opaque	n.d.
R1-16Δ*traI*	−	−	None	n.d.
R1-16Δ*traI*	R1 TraI	++	Opaque	5.3 × 10^11^
R1	−	−	None	n.d.
pOX38	−	+++	Clear	1.3 × 10^14^
pOX38*traD411*	−	−	None	n.d.
pOX38MK3	−	+++	Clear	1.8 × 10^14^
pOX38Δ*traI*	−	++	Opaque	1.2 × 10^14^

DNA requirements				

Plasmid	Coresident plasmid^a^	Infection level^b^	Plaque morphology	pfu ml−1^c^
R1-16	−	+++	Clear	1.4 × 109
R1-16	R1 *oriT* plasmid	+	Very opaque	n.d.
R1-16Δ*nic*	−	−	None	n.d.
R1-16Δ*oriT*	−	−	None	n.d.

a Vector alone had no effect (not shown).

b +++, wild-type; ++, opaque but countable; +, very opaque and not countable; −, none.

c Plaque forming units per millilitre; n.d., not detected.

In comparison, phage sensitivity of cells carrying the F derivative pOX38, or mutants thereof (Table 1), confirmed a dependence on *traD* (Schoulaker and Engelberg-Kulka, 1978) but was independent of *traI*. The pOX38MK3 derivative also supported efficient plaque formation. Taken together, the sum of our initial work demonstrated that, in contrast to F-carrying cells, phage sensitivity conferred by R1-16 requires *traI* and activities supported by *oriT*, including, presumably, assembly of the relaxosome. The basis of this plasmid-specific difference may have to do with the system-specific nature of the F and R1 relaxosomes as well as marked differences in the C-terminal extensions of the two TraD proteins involved in relaxosome docking. Nonetheless, the strong phenotypes observed with the R1 model provide a means to investigate the mechanisms of R17 nucleoprotein uptake.

### A TraI fragment including the relaxase domain and TSA is sufficient for cellular uptake of R17 RNA–protein A

**Fig. 2:**
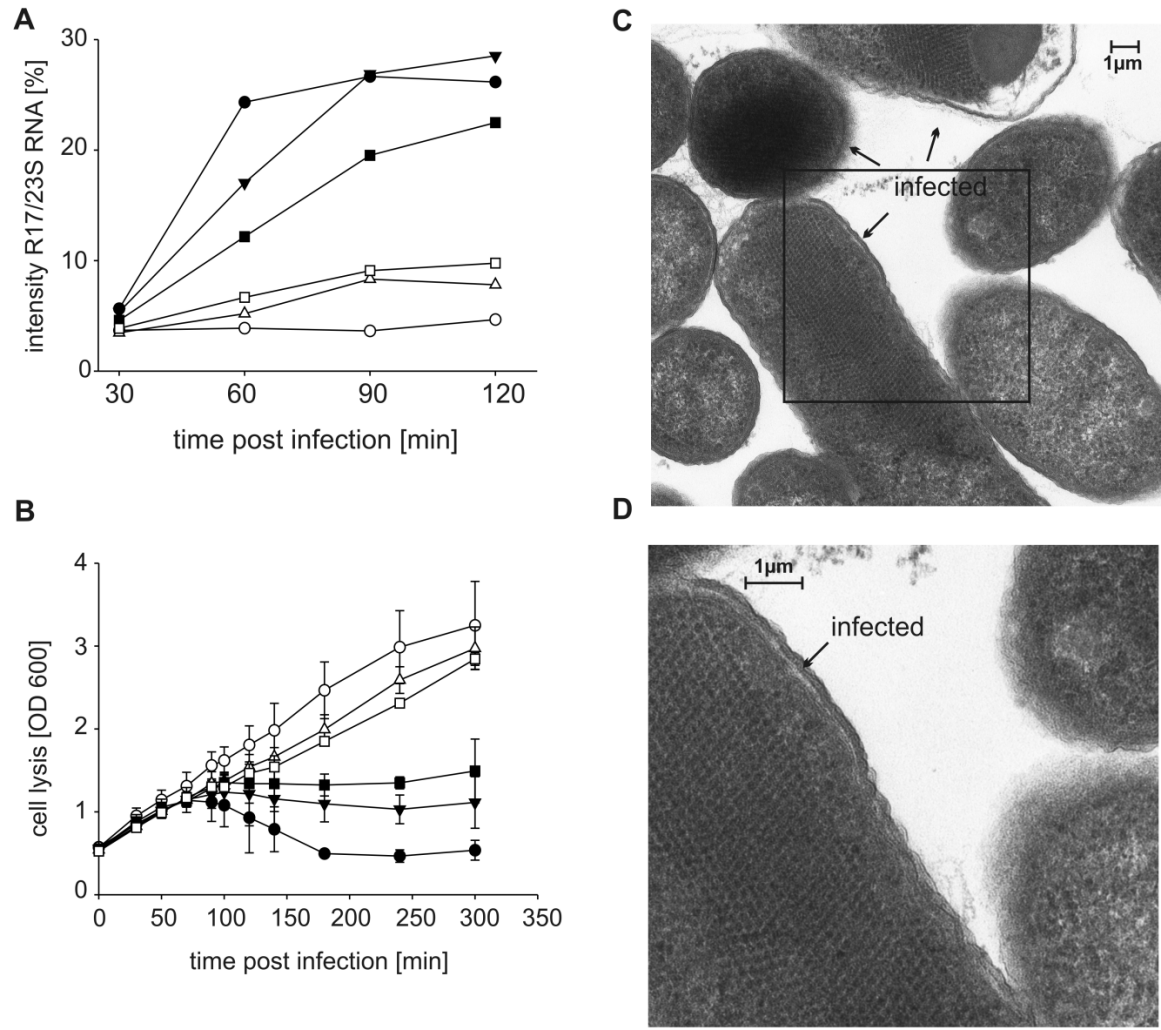
TraI fragments including the relaxase domain and TSA are necessary and sufficient for nucleoprotein uptake. R17 RNA yield (A) and phage-induced cell lysis (B) are shown for plasmid R1-16 (•) or the R1-16Δ*traI* (○) derivative when complemented with a wild-type R1 *traI* allele (▾) or fragments thereof. High RNA yields and cell lysis were observed with TraI N_1-992_ (relaxase + TSA) (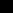). No complementation was observed with a combination of TraI fragments N_1-309_ (relaxase) and N_310-1756_ (helicase) (▵) or fragment N_310-1756_ alone (□). Values represent the mean of at least three experiments. Standard deviations are shown. Complementation of R1-16Δ*traI* with TraI N_1-992_ supports normal progression of phage replication as shown by electron microscopy of the infected cells (*arrows*) (C). In D an enlarged region of the distinctive honeycomb pattern of R17 is highlighted.

We showed that host cell R17^S^ required a TraD protein with an intact NTP binding site and TraI. The ability to complement the TraI function *in trans* enabled us to ask which domains of TraI are required for efficient nucleoprotein uptake. A very detailed functional map of F-like TraI proteins is available, as well as classes of well-defined mutations (Fig. 1B) (Haft *et al*., 2006; Mihajlovic *et al*., 2009; Sut *et al*., 2009; Dostal and Schildbach, 2010; Dostal *et al*., 2010; Lang *et al*., 2010; Wright *et al*., 2011). Cultures of *E. coli* host cells carrying R1-16Δ*traI* and expressing wild-type or truncated alleles of *traI in trans* were infected with R17 phage. On the population level we visualized the progress of R17 RNA replication using agarose gel electrophoresis, and detected host cell lysis by monitoring the optical density of infected cultures (Fig. 2A and B respectively). We also used transmission electron microscopy to routinely confirm a normal progression of phage replication in single cells. Suspensions of identical hosts were exposed to phage then fixed in agar. Ultrathin sections of these blocks were prepared to reveal the cytosolic contents of individual cells from various levels. Intracellular R17 phage are readily visible as single particles that form a distinct honeycomb pattern (as illustrated in Fig. 2C and D). The cells we routinely observed were either full of hundreds of visible phage particles or lacked these altogether. We found no evidence for any mutant under any condition to indicate that the host population was uniformly infected but was delayed or dysfunctional in phage replication. We conclude therefore, that the requirement for TraD and relaxosome components observed in this study is manifest on the level of RNA entry.

Dependence of these processes on *traI* is shown (Fig. 2). Coexpression of a combination of TraI fragments N_1-309_ (relaxase) and N_310-1756_ (helicase), or fragment N_310-1756_ alone failed to complement the infection phenotype of R1-16Δ*traI* (Fig. 2; open symbols), just as these fragments fail to complement R1-16Δ*traI* for conjugation (Lang *et al*., 2010). By comparison high RNA yields (Fig. 2A) and cell lysis (Fig. 2B) were observed with the full-length TraI. Remarkably, TraI N_1-992_ alone was also sufficient to reconstitute phage propagation (3.6 × 10^11^ pfu ml^−1^) that was also apparent by the abundant production of R17 RNA (Fig. 2A) and the arrest of host cell culture density beginning 100 min post infection (Fig. 2B). TraI N_1-992_ contains the T4CP docking position TSA physically linked to the relaxase and ssDNA binding domains (Fig. 1B). This is the same fragment required – in combination with the entire helicase fragment (TraI N_310-1756_) – for effective conjugative gene transfer (Lang *et al*., 2010). Importantly, for RNA uptake, however, the helicase and C-terminal TraM interacting domains on fragment TraI N_310-1756_ are completely dispensable.

TraI N_310-1756_ carries two TS and our earlier work showed that even though this protein cannot form the TraI-T DNA adduct at *nic* typical for the full-length secretion substrate, it can be translocated to recipient cells (Lang *et al*., 2010). Thus docking of TraI N_310-1756_ to TraD should be normal. Nonetheless, that reaction alone is clearly not sufficient to support initiation of the phage nucleoprotein import process. Figure 2 illustrates the absence of R17 RNA synthesis in culture when R1-16Δ*traI* hosts express TraI N_310-1756_ (Fig. 2A) and the continuous growth of cells after addition of phage similar to that of hosts carrying R1-16Δ*traI* alone (Fig. 2B). In agreement with our earlier observations in conjugative transfer (Lang *et al*., 2010), the requirement for TraI in R17^S^ was only met when the protein's N-terminal relaxase and ssDNA binding domains are physically linked to TSA. We propose that interactions between TraD and TraI TSA are communicated over this arm of the protein to the relaxase bound at *oriT.* The flow of regulatory signals appears to be crucial to early steps in both nucleoprotein uptake and secretion. We further propose that this bidirectional process alters both the conformation and the activities of the T4CP, as well as the relaxosome, in a manner that depends on *oriT* DNA. The observation that R1-16Δ*nic* and R1-16Δ*oriT* do not support phage infection, despite the presence of all proteins, is consistent with this hypothesis.

### TraI N_1-992_-catalysed *nic*-cleavage is stimulated by TraD

**Fig. 3:**
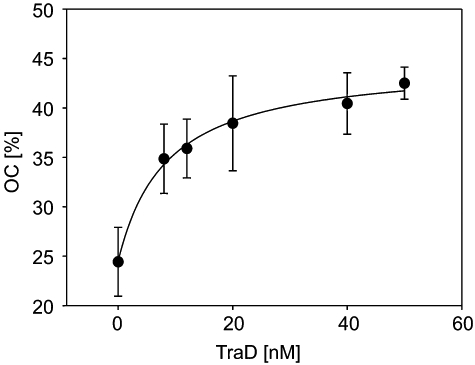
TraI N_1-992_-catalysed *nic*-cleavage is stimulated by TraD. Conversion of supercoiled *oriT* DNA to the open circular (OC) form by purified TraI fragment N_1-992_ is expressed as per cent of total DNA substrate. *Nic*-cleavage was measured with 100 nM TraI N_1-992_ alone or with increasing concentrations of TraD. Values represent the mean of at least three experiments. Standard deviations are shown.

If the proposed model is true, it is reasonable to expect that interaction between TraD and this functional arm of TraI would affect the DNA processing reactions it catalyses. We showed previously that the purified cytosolic form of TraD enhanced TraI-catalysed *nic*-cleavage *in vitro* but, consistent with its lack of TS docking domains, the isolated relaxase TraI N_1-309_ was not stimulated by TraD (Mihajlovic *et al*., 2009). Here we purified the TraI N_1-992_ fragment and assayed for biochemical modulation of the *nic*-cleavage reaction (Fig. 3). As predicted, the activity of TraI N_1-992_ in this reaction was stimulated by TraD in a dose-dependent manner.

### Catalytically active relaxase and high affinity interaction with *nic* DNA is required for efficient nucleoprotein uptake

**Fig. 4:**
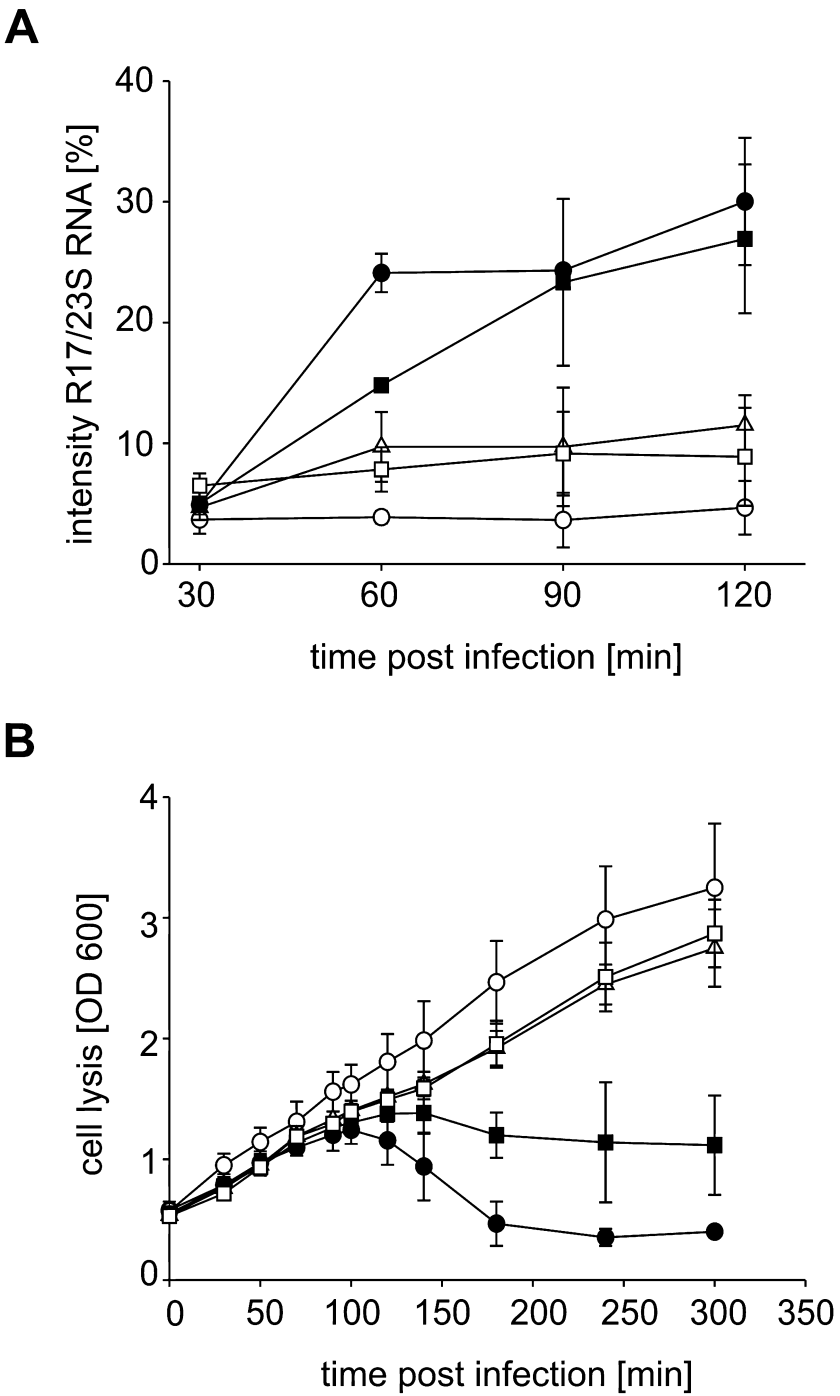
Relaxase catalysis and high affinity interaction at *nic* is required for efficient nucleoprotein uptake. R17 RNA yield (A) and phage-induced cell lysis (B) are shown for plasmid R1-16 (•) and R1-16Δ*traI* (○) complemented with TraI N_1-992_ (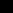). In contrast no complementation was monitored for the mutant TraI N_1-992_ variants, which are cleavage deficient (□) or show a reduced affinity for *oriT*-specific sequences (▵). Values represent the mean of at least three experiments. Standard deviations are shown.

To help us to understand the reactions involved in nucleoprotein uptake and their control, various mutant forms of TraI N_1-992_ were tested. Dostal and Schildbach demonstrated that replacement of the relaxase catalytic tyrosine in F plasmid TraI eliminates nicking activity on ssDNA (Dostal *et al*., 2010). We replaced Y16F and Y17F on the truncated TraI N_1-992_. We then created a mutant allele that should alter the protein's high affinity interactions with ssDNA that are associated with the relaxase. We again drew on earlier data from the Schildbach group showing the importance of specific amino acids to this activity in the F system (Harley and Schildbach, 2003). Given that the wild-type sequences surrounding *nic* are identical in the R1 and F TraI binding sites, we recreated in TraIN_1-992_ three amino acid exchanges E153D/Q193R/R201Q previously characterized by the Schildbach group. These mutations were reported to substantially reduce the affinity of relaxase-associated interactions with ssDNA but not completely eliminate cleavage at *nic*. The new alleles were tested for complementation of the *traI* requirement in a phage infection analysis (Fig. 4). In this experiment R17 RNA yield and phage-induced cell lysis supported by TraI N_1-992_ and R1-16Δ*traI* were in good agreement with those of plasmid R1-16. In contrast no complementation was observed with the cleavage deficient variant or the E153D/Q193R/R201Q mutant protein.

### A full-length TraI lacking relaxase-associated ssDNA binding activity maintains helicase activity

**Fig. 5:**
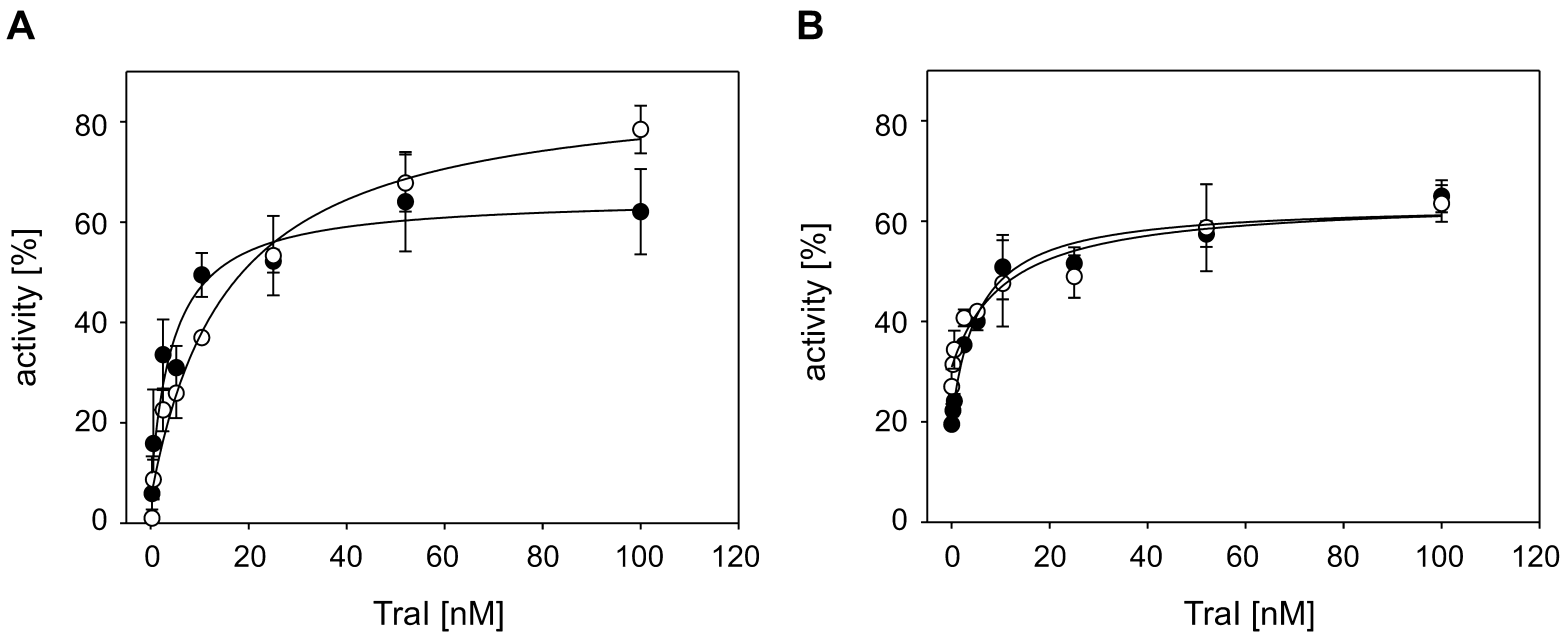
Absence of relaxase-associated ssDNA binding relieves sequence-specific inhibition of *oriT* unwinding. Duplex unwinding catalysed by increasing concentrations of TraI (•) and TraI M2L/E153D/Q193R/R201Q (○) on heteroduplex *oriT* substrates that present *nic* and the IR for TraI binding as ssDNA (A), or constrained in dsDNA (B). The indicated per cent of substrate unwound represents the mean of three independent assays. Standard deviations are shown.

The amino acid exchanges introduced to TraI N_1-992_ were chosen because these are known to substantially reduce the affinity of the protein for sequences surrounding *nic* (Harley and Schildbach, 2003). Reduced affinity of the relaxase-associated binding site was confirmed for a truncated TraI N_1-330_ M2L/E153D/Q193R/R201Q (K. Guja and J. Schildbach, unpublished), but longer versions of the protein have not been analysed. To verify that a longer form of the mutant protein is not globally disrupted, we purified a full-length variant TraI and checked simultaneously several of its activities: DNA unwinding, T-strand cleavage and negative cooperativity between ssDNA binding sites, using an experimental system described previously (Csitkovits *et al*., 2004; Sut *et al*., 2009). The test substrates present a bubble of open duplex to support helicase loading onto ssDNA. The position of *oriT* DNA in single-stranded conformation centres either on the conserved IR of R1 and F, which supports high affinity ssDNA binding by the relaxase associated site of TraI (Williams and Schildbach, 2006), or a sequence outside of *nic* (G2028), which lacks those ssDNA recognition features (Sut *et al*., 2009). As expected the mutant protein exhibited robust helicase activity on both substrates (Fig. 5A and B). The activity of both proteins was identical on a substrate lacking *oriT*-specific sequence in single-stranded form (Fig. 5B). By contrast, wild-type TraI has lower unwinding activity on IR substrates than the mutant TraI (Fig. 5A). We believe that the sequence-specific inhibition of wild-type TraI (Sut *et al*., 2009) results from the negative cooperativity regulating the relaxase- and helicase-associated ssDNA binding sites (Dostal and Schildbach, 2010). The helicase activity of mutant TraI was higher than wild-type on IR DNA. This result is expected for a mutant protein deficient in ssDNA binding via the relaxase-associated site. The helicase activity of the mutant should be higher than wild-type on *nic*-specific DNA since the negative cooperativity that normally occurs between binding sites would be reduced in the mutant protein. The assay provides an important control confirming that TraI functions involving the central region of the mutant protein are intact. We conclude that failure of the TraI N_1-992_ E153D/Q193R/R201Q to support phage sensitivity is due to poor relaxase affinity for the R1 *nic* sequence.

### TraI TSA but no helicase-associated activities are necessary for efficient nucleoprotein uptake

**Fig. 6:**
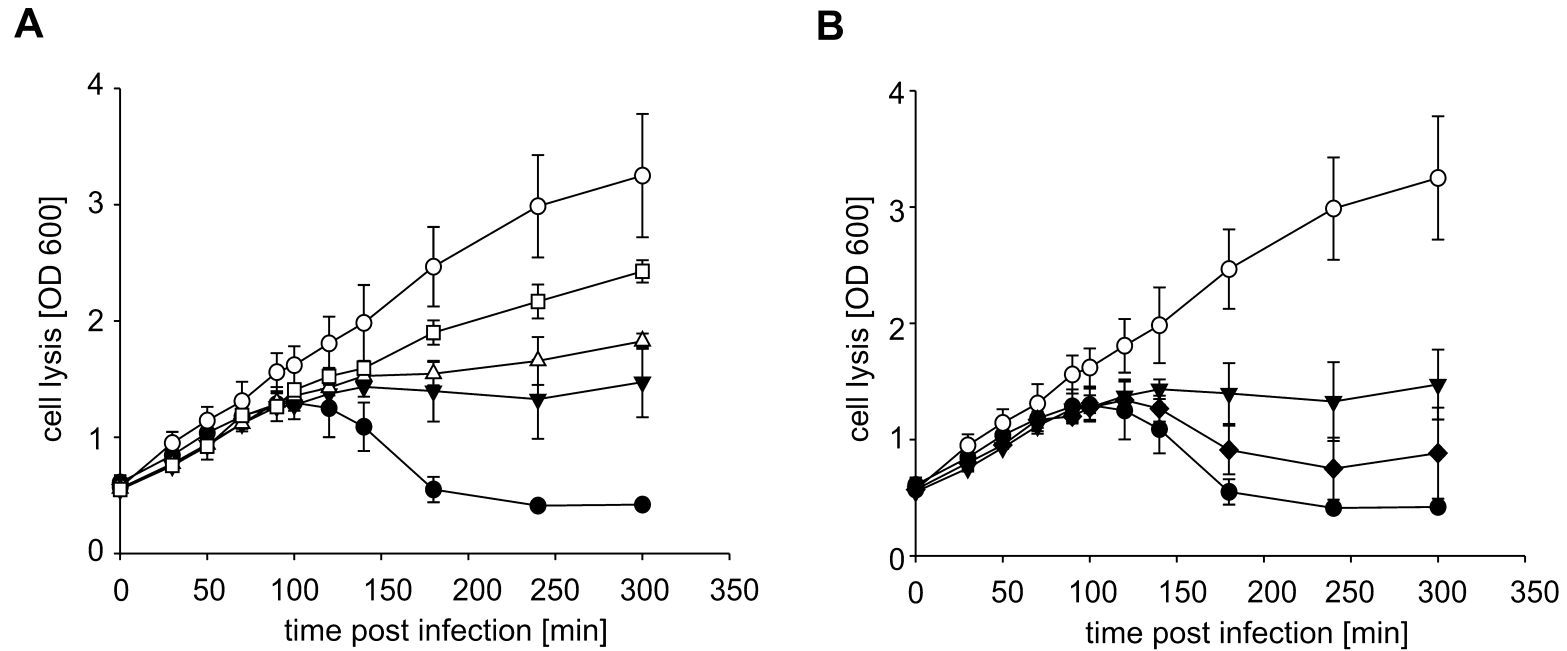
TraI TSA but not helicase-associated activities are necessary for efficient nucleoprotein uptake. A and B. Phage-induced cell lysis was monitored for plasmid R1-16 (•) or the R1-16Δ*traI* without (○) or with a wild-type *traI_F_* allele (▾) or insertion mutants (i31) thereof. *traI_F_* alleles with the insertion in TSA (TraIi593, ▵; TraIi681, □) complement the R1-16Δ*traI* defect poorly (A), while *traI_F_* variant TraIi369 (♦), carrying a normal TSA, complements efficiently (B). Values represent the mean of at least three experiments. Standard deviations are shown.

We next investigated 31 residue insertion derivatives of TraI from plasmid F [TraIi (position of insertion); created by B. Traxler (Haft *et al*., 2006)]. These were selected because the site of insertion is known to disrupt the helicase-associated ssDNA binding site of TraI and reduced negative cooperativity between the sites [TraIi369, TraIi593 and TraIi681 (Dostal and Schildbach, 2010)]. Moreover, we compared those where the site of insertion fell within TSA – possibly compromising efficient contact with TraD (TraIi593, TraIi681) – with TraIi369 where TSA is not directly affected (Lang *et al*., 2010). Phage-induced cell lysis was monitored for hosts carrying R1-16Δ*traI* with either the wild-type *traI_F_* allele or the insertion mutants (Fig. 6). The *traI_F_* allele complemented the lack of *traI_R1_* efficiently, whereas both variants with disrupted TSA (TraIi593, TraIi681) did not. In contrast, effective complementation was observed with the *traI_F_* variant encoding wild-type TSA (TraIi369). Since this mutation also reduces affinity for ssDNA by the helicase-associated site and exhibits significantly reduced negative cooperativity of binding, we conclude that these activities of the wild-type protein are not important to regulation of RNA entry.

In summary, TraI N_1-992_ is sufficient to provide a regulatory function required in host cells for efficient R17 nucleoprotein uptake. Our results pinpoint the relevant features of this functional domain to include high affinity interactions of the relaxase with ssDNA, the relaxase catalytic tyrosine, and TSA carried on the same polypeptide. Remarkably, TraI of plasmid F can provide this regulatory activity for the R1-16 machinery even though R17 uptake by the T4SS of F does not require TraI. Together, these data imply that the novel activity for TraI contributes to a larger docking and activation process involving TraD, *oriT* and possibly other components of the R1-16 T4SS.

## Discussion

‘Male-specific’ filamentous and RNA phage exploit the presence of F-like conjugative pili and the underlying envelope spanning transport machinery to gain entry to bacterial cells. Sensitivity to distinct phage groups has been instrumental in classifying pilus types (Frost *et al*., 1985). Individual phage also exhibit exquisite discrimination of natural and induced variations in pilin proteins, which helped establish structure–function relationships in pilin biochemistry (Frost *et al*., 1985; Frost and Paranchych, 1988; Manchak *et al*., 2002). The route of entry taken by the R17 phage used in this study has not been determined. The tubular nature of the F-pilus offers a passive conduit for uptake, as proposed by Brinton (Brinton, 1965). Alternatively, the dynamic cycles of F pilus outgrowth and disassembly may be involved. Phage adsorption does not induce pilus retraction, but once triggered, the process draws adsorbed phage to the cell surface (Clarke *et al*., 2008). F-pilin subunits of retracting pili re-enter the membrane pool. The central structure of T4S components is likely to remain, and may provide the phage access to the host cell cytosol. The T4CP TraD of F-like plasmids is not involved in pilus biogenesis but is essential for host sensitivity to group I RNA phages R17, f2 and MS2, but not Qβ (Valentine *et al*., 1969; Schoulaker and Engelberg-Kulka, 1978). Based on what we now know about the decisive role T4CPs play in connecting the secretion channel with the cytoplasm and in recruiting and initiating (nucleo)protein secretion, further investigation of the T4CP-dependent phage infection process is warranted.

Here we develop the R17 nucleoprotein uptake model using the IncFII plasmid R1. We confirm the requirement for the T4CP and demonstrate that mutation of its NTP binding site eliminates function. We also demonstrate that the R1 relaxosome is involved in an essential manner. A novel functional domain was delineated within TraI N_1-992_ that is necessary and sufficient to support phage uptake by R1-16Δ*traI*. The mechanism involved requires high affinity interactions of the relaxase with ssDNA, the relaxase catalytic tyrosine and the TSA region of the protein that interacts specifically with TraD. R17^S^ was eliminated also by deletion of DNA that is important to relaxosome assembly or specific DNA recognition by the TraI relaxase. Thus a ternary complex of plasmid *oriT*, TraI N_1-992_ and TraD provides the essential activity. Normally, TraY and TraM are also bound to *oriT* and TraM engages in highly specific interactions with the C-terminus of TraD (Disque-Kochem and Dreiseikelmann, 1997; Beranek *et al*., 2004; Lu *et al*., 2008; Wong *et al*., 2011). The negative effects of *traM* and *traY* deletion on R17^S^ could not be complemented, but we anticipate that this larger complex is involved in the nucleoprotein import mechanism. A simple binding or docking interaction between the relaxosome and the T4CP is not adequate to describe the activity, as shown by the TraI tyrosine exchange, and TraDK198T variants. What then is the subcomplex doing? T4 secretion typically depends on signals originating from contacts between cells. We showed earlier that the TraI N_1-992_ activating domain is sufficient to initiate plasmid DNA transfer as long as the helicase domain is provided separately (Lang *et al*., 2010). Taken together these data support a simple explanation where the docked complex of relaxosome and T4CP is the receptor for signals transmitted to the cell interior. Further the initial mechanisms that process the incoming signals into activation of both TraD-mediated nucleoprotein import and export processes are conserved.

**Fig. 7:**
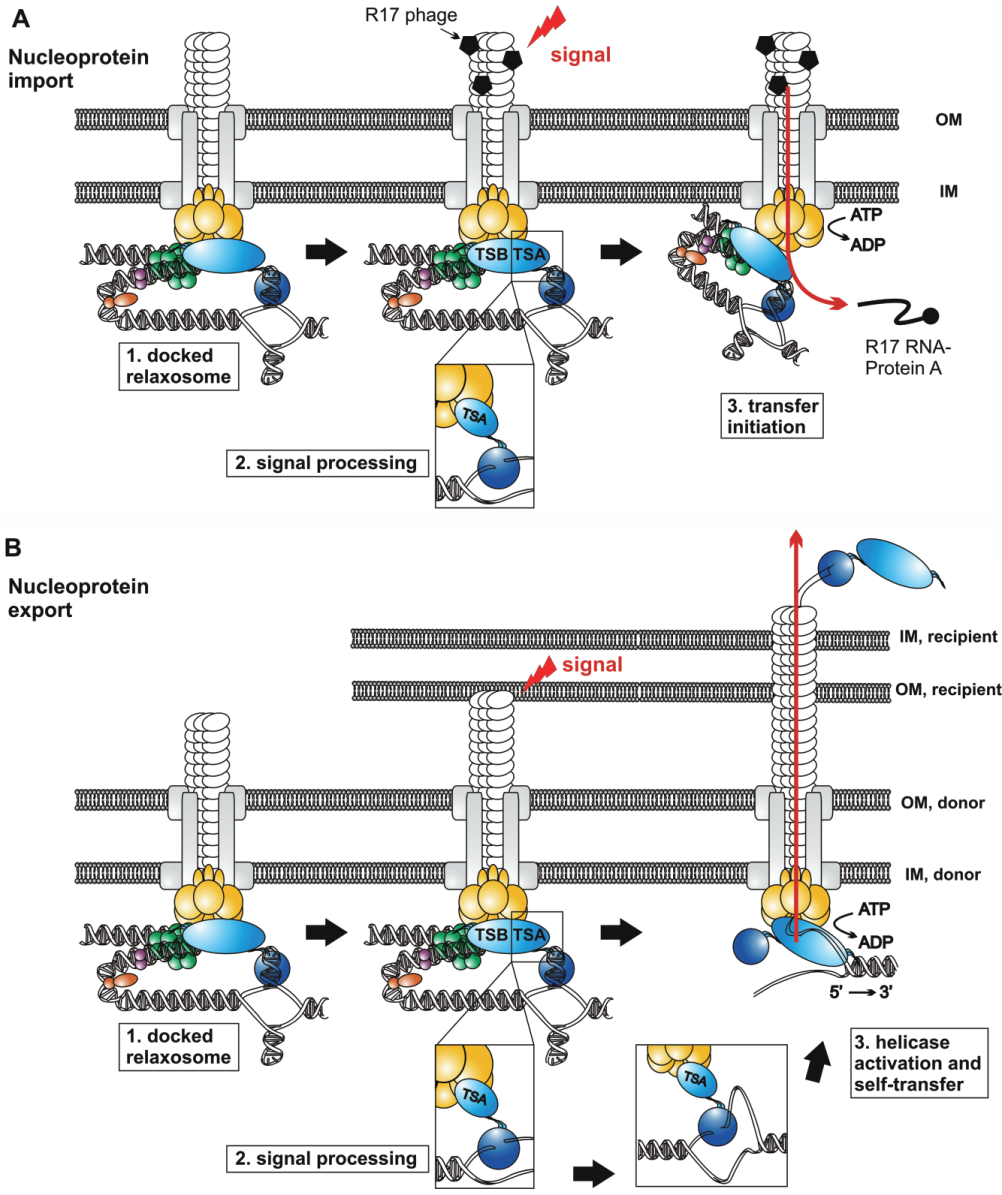
Stages of T4 nucleoprotein transfer initiation mediated by plasmid R1-16. A and B. The T4 transfer apparatus is constitutively expressed and assembled. Stage 1: The relaxosome, containing *oriT* bound by TraM (*green*), TraY (*violet*), IHF (*orange*) and TraI (*blue*) is docked to the T4CP (*yellow*) via TraI translocation signals (TSA, TSB) and TraM binding. Stage 2: Productive pilus contacts with adherent R17 phage (A) or another cell (B) produces signals (*lightning bolt*) conveyed over the pilus to the T4CP-relaxosome receptor. Processing of those distinct signals into nucleoprotein translocation requires at a minimum, a conserved relaxase activation domain. TraI N_1-992_ comprises this domain for R1 (*inset*). Stage 3: During phage infection (A) downstream activation of the T4CP ATPase activity is essential for uptake of the R17 RNA–protein A complex. Alternatively, when cell contact permits plasmid self-transfer (B) initiation still requires productive interactions between the T4CP and R1 TraM bound to *oriT*. Signal processing is performed by the relaxase activation domain occupying the T4CP sites (*inset*). If TraI is bound to TraD, then the TraI N_1-992_ domain converts the cell contact signal into localized *oriT* melting (*inset*) and helicase activation in a separate downstream step.

In this model (Fig. 7) TraD is anchored to the base of the transfer channel while its cytosolic domain binds TraM, TraI and *oriT* DNA (Stage 1). The relaxosome is catalytically active at *nic* in the absence of TraD, but cleavage is stimulated by its presence (Mihajlovic *et al*., 2009). NTP hydrolysis by TraD appears to be silent. Progression from this stage requires signals communicated over the pilus from the cell exterior (Stage 2). In the case of R17 phage adsorption the productive receptor for the incoming signal is TraD docked by the R1 relaxosome (Fig. 7A). The accessory factors bound at *oriT* are important, but the key component is the TraI N_1-992_ docking and activation domain (*inset*). We propose that processing of the external signal through this mechanism depends on the physical link between catalytic activity at the plasmid nick site and the T4CP to modulate TraD conformation and thereby activate the essential ATPase. The activation initiates translocation of the RNA–protein A complex into the host cell (Stage 3). Activities related to TraI helicase and its C-terminal domain are dispensable. The fact that this form of translocation activation is independent of the helicase domain and that the helicase itself is not activated on the docked *oriT* under these conditions is logical, since phage penetration would otherwise result in plasmid DNA being extruded pointlessly into the medium. This has never been observed.

In the case that the assembled pilus contacts a suitable recipient cell (Fig. 7B), the productive receptor for the incoming signal is again TraD docked by the R1 relaxosome. TraI N_1-992_ is fully capable of supporting initiation reactions to this point, but secretion of R1 requires a final unique step of helicase activation. Duplex unwinding can be catalysed *in vitro* by the truncated domain TraI N_310-1756_ or the full-length protein. However, the truncated helicase only supports transfer when combined with TraI N_1-992_, but not with the relaxase lacking TSA (TraI N_1-309_) (Lang *et al*., 2010). It follows, therefore, that the progression of activation steps when TraI N_1-992_ is docked to TraD also induces the localized denaturation of *oriT* DNA (Csitkovits *et al*., 2004) necessary to load and initiate helicase activity. The interaction of TraD and helicase stimulates DNA unwinding (Sut *et al*., 2009). In summary, we propose that the initiation cascades induced by these distinct pilus mediated stimuli progress through parallel steps mediated by identical components of the R1 machinery. The ultimate difference is that the phage mediated signals repress, or cannot induce, the final step of helicase activation. That modulation through evolution supports the survival of both the phage and the plasmid.

**Fig. 8:**
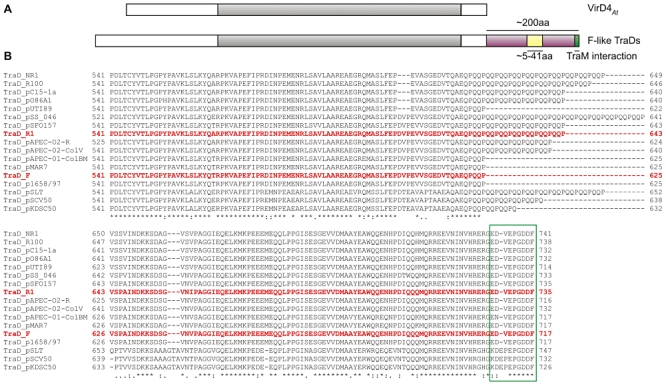
F-like T4CPs display C-terminal heterogeneity. A. The molecular architecture of VirD4 from *A. tumefaciens* is compared to F-like TraD proteins according to Alvarez-Martinez and Christie (2009). The conserved NTP binding domains are indicated in *grey*. The TraD C-terminal extension (∼ 200 residues, *violet*) harbours a variable region of glutamine proline sequential repeats (QQP motif, *yellow*) and the TraM interaction domain (*green*). B. The sequence alignment was performed by using the ClustalW2 multiple sequence alignment program (http://www.ebi.ac.uk/Tools/msa/clustalw2/). Only the last ∼ 200 residues of the aligned T4CPs are shown. T4CPs of plasmid R1 and F, investigated in this study, are highlighted in red. The TraM interaction domain according to Lu *et al*. (2008) is shown (*green box*). The T4CP designations include the protein names followed by the plasmid name, according to the GenBank database. Accession numbers for T4CPs are: YP_001096500 for TraD_NR1, NP_052980 for TraD_R100, NP_957631 for TraD_pC15-1a, YP_788090 for TraD_pO86A1, YP_538736 for TraD_pUTI89, YP_313445 for TraD_pSS_046, YP_001294757 for TraD_pSFO157, AAT85682 for TraD_R1, YP_190116 for TraD_pAPEC-02-R, YP_443957 for TraD_pAPEC_02_ColV, YP_001481211 for TraD_pAPEC-01-ColBM, ABG29548 for TraD_pMAR7, BAA97972 for TraD_F, NP_862947 for TraD_p1658/97, NP_490588 for TraD_pSLT, YP_209289 for TraD_pSCV50, NP_073256 for TraD_pKDSC50.

The results of this study raise a number of interesting points concerning the coevolution of F plasmids and the phage that target conjugative pili. Given that the phage life cycle destroys their mutual host, the evolutionary interplay can be likened to an arms race. Conjugative plasmids are subject to strong selection for mutations that confer phage resistance yet allow conjugation. Natural variation in TraA pilins alters phage sensitivity and five types are known among related F plasmids (represented by F, ColB2, R1-19, R100-1 and pED208) (Frost *et al*., 1985; 1994; Anthony *et al*., 1999; Manchak *et al*., 2002). Rapid selection for compensatory mutations among the phage population inevitably follows. The Paranchych laboratory noted that only 50% of a given preparation of R17 phage was active for pilus attachment, conceivably as a result of emerging mutations (Paranchych *et al*., 1970). In this study of R1-16, the process of R17 phage penetration was connected to assembly and function of the conjugative relaxosome in conjunction with the T4CP. It would appear that only T4SS that are maximally prepared for conjugative plasmid transfer are also vulnerable to phage uptake. This stringency benefits the conjugative plasmid. The requirement for the relaxosome in R17 exploitation of the R1 T4SS was not shared by the F derivative pOX38. The TraI_F_ protein could provide that essential function with R1-16 even though it is not inherent to the infection mechanism via the cognate T4SS. This distinction suggests that related F plasmids may have diversified on the level of relaxosome and particularly the T4CP in response to phage-driven selection. T4CPs are generally conserved in the NTP binding domain but display variability at the N- and C-terminal domains (Alvarez-Martinez and Christie, 2009). The C-terminal extensions of TraD proteins of F-like plasmids range from 150 to 200 amino acids in length (Fig. 8). The unstructured extension is important to the specificity of substrate interaction and forms extensive contacts to TraM tetramers (Sastre *et al*., 1998; Lu *et al*., 2008; Wong *et al*., 2011). Perhaps, analogous to the VirB4-like protein TrwK, the C-terminal extension may also regulate ATPase activity (Pena *et al*., 2011). The TraD proteins of related plasmids display heterogeneity in the C-terminal extension including the length of a variable region of glutamine proline sequential repeats (QQP motif). TraD_F_ carries just five residues (QPQQP) whereas TraD_R1_ contains 23. The mechanistic basis of the plasmid-specific differences revealed in this study may arise through variation in TraD. This possible explanation is under investigation. Our findings further suggest that an important factor driving diversification among the F-like T4CPs is phage resistance.

## Experimental procedures

### Strains and plasmids

All *E. coli* K-12 strains used in this study are described in Table S1. Plasmids are described in Table S2.

### DNA preparation and PCR amplification

Plasmid DNA was purified from *E. coli* cells with the QIAprep Spin Miniprep Kit (Qiagen, Hilden, Germany). Restriction endonucleases and DNA modifying enzymes were obtained from Fermentas GmbH (St. Leon-Rot, Germany). DNA fragments for cloning were amplified using Phusion High-Fidelity DNA Polymerase (Finnzymes Oy, Espoo, Finland) or the Taq-Polymerase (New England Biolabs, Beverly, MA, USA). The correct DNA sequence of all amplified fragments used in cloning was verified. Enzymes were used according to manufacturers' recommendations.

### Construction of *traY*, *nic* and *oriT* null derivatives

Primer sequences are shown in Table S3. To generate R1-16Δ*traY*, R1-16Δ*nic* and R1-16Δ*oriT*, the primer pairs traYko1_FW and traYko1_Rev, oriTko1_FW and oriTko1_Rev, or oriTko1_FW and oriTko2_Rev were used to amplify a *loxP*-TetRA-*loxP* cassette from CSH26Cm::LTL (Lang *et al*., 2010). The amplified fragments were introduced into *E. coli* DY330 [R1-16] and integrated via homologous recombination as previously described (Reisner *et al*., 2002). Introduction of the CFP B plasmid into strains carrying these null derivatives catalysed a Cre/*loxP* mediated recombination reaction excising the *tetRA* cassette.

### Construction of expression plasmids

The inserts for pMSTraD_wt and pET29TraI(1–992) were amplified from R1-16 with the indicated primers (Tables S2 and S3) and ligated with pMS119EH or pET29a respectively. pET24a-TraI carries wild-type F *traI* while pET24a-TraI^M2L/E153D/Q193R/R201Q^ expresses just the 36 kDa relaxase fragment of F TraI with the indicated mutations (both kindly provided by J. Schildbach, Johns Hopkins University). Reduced affinity of the mutant variant for ssDNA was confirmed by K. Guja and J. Schildbach (unpublished results). Both plasmids were cut with NdeI and StuI to introduce the mutant fragment of pET24a-TraI^M2L/E153D/Q193R/R201Q^ into the full-length allele, resulting in pCG03. Two-step PCR was used to generate pMSTraD_A, and pRelTSA^Y16FY17F^. In the first step primer sets 1 and 2 (Tables S2 and S3) were used to amplify two fragments from R1-16, which both carried the desired point mutations. In the second step these two fragments were annealed and amplified with primer set 3. The fragments were cut with EcoRI/HindIII or EcoRI/BamHI and religated with pMS119EH or pGZ119EH respectively. Two-step PCR was also used to generate pRelTSAFR100. The first fragment carried the mutated F *traI* relaxase domain from pCG03 and the second carrying bp 927–2976 from pCG02. Annealing, amplification with primer set 3 and ligation with *Eco*RI/*Bam*HI cut pGZ119EH generated pRELTSAFR100 expressing the chimeric E153D/Q193R/R201Q TraI_1-992_.

### Protein purification

TraDΔN130 was expressed and purified as described previously (Mihajlovic *et al*., 2009). TraI M2L/E153D/Q193R/R201Q was expressed and purified as described previously for full-length R1 TraI (Mihajlovic *et al*., 2009).

*Escherichia coli* BL21(DE3) [pet29TraI_1-992_] was grown in 1 l LB media supplemented with 40 µg ml^−1^ kanamycin to an A_600_ 0.6. Overexpression was induced by addition of isopropyl-1-thio-α-d-galactopyranoside (IPTG) to a final concentration of 1 mM. Cells were harvested after 5 h shaking at 37°C and pellets were frozen at −80°C. Frozen cells were thawed overnight at 4°C. The pellet was resuspended in 15 ml of buffer I [50 mM sodium phosphate, 250 mM sodium chloride, 10 mM imidazole, 0.02% sodium azide (w/v), pH 8] and lysed by two passages in a French press cell. The cytoplasmic fraction was obtained by centrifugation at 21 000 *g* for 1 h. The supernatant was filtered (0.4 µm) and applied to a 10 ml His-Select Nickel affinity gel, equilibrated with buffer I. Adsorbed proteins were eluted with a 60 ml gradient of 0.01–0.5 M imidazole in buffer I. Fractions containing the protein were pooled and dialysed at 4°C overnight against a 100-fold volume of buffer II (50 mM sodium phosphate, 1 mM EDTA, 100 mM NaCl, pH 7.5). Soluble ammonium sulphate (AS) was added to a final concentration of 1 M to the dialysate and this fraction was loaded on two 5 ml HiTrap Phenyl HP columns, connected in tandem and equilibrated with buffer II plus 1 M AS. The column was developed with a 180 ml decreasing gradient of 1 to 0 M AS in buffer II. Peak fractions eluting between 300 and 150 mM AS were dialysed at 4°C against a 100-fold volume of buffer III [50 mM sodium phosphate, 100 mM NaCl, 0.02% sodium azide (w/v), pH 7.5], supplemented to 40% glycerol, concentrated with a Amicon filter device (Millipore) and then stored at −80°C. The apparent molecular mass of the protein of 110 kDa was confirmed by Coomassie blue staining following denaturating polyacrylamide gel electrophoresis.

### R17 lysate preparation

To prepare fresh phage lysate 2 ml of an overnight culture of MS411 [R1-16] was pelleted at 4000 *g* for 8 min and then suspended in 1 ml 10 mM MgSO_4_. One hundred microlitres of cell suspension were mixed with an equal volume of R17 phage lysate (*c*. 10^9^ pfu) in appropriate dilutions in ice-cold TMG-buffer [10 mM Tris-HCl, pH 7.4, 5 mM MgSO_4_, 0.01% (w/v) gelatine]. The phage–cell mixture was incubated for 5 min at room temperature. After incubation, 3 ml of R-top agar containing 10 mM MgSO_4_, 2 mM CaCl_2_ and 5 mM glucose were gently mixed with 200 µl of the cell–phage suspension and spread onto pre-warmed LB agar plates containing the appropriate antibiotics. Plates were incubated for at least 6 h at 37°C until plaque formation was visible. Two-millilitre ice-cold TMG-buffer was added onto plates showing confluent lysis and plates were kept at 4°C for 5 min. Top-agar and TMG-buffer was scraped off the plates, transferred to a 50 ml tube, and then centrifuged at 4000 *g* for 10 min. The supernatant containing the R17 phage lysate was stored at 4°C.

### Infection studies with the male-specific phages

Plaque assays were performed with *E. coli* MS411 harbouring the desired plasmids (Table 1) under the conditions described for the phage lysate above, except that plates were incubated overnight at 37°C. Liquid infection assays were performed as described previously (Bayer *et al*., 1995). Briefly, 40 ml LB medium containing 2 mM CaCl_2_ and the appropriate antibiotics were inoculated to A_600_ 0.05 with the desired strain. The cells were grown at 37°C to an A_600_ 0.6 and 4 ml R17 phage lysate was added. Cultures were grown at 37°C with shaking and cell lysis was determined by measuring the A_600_ at the indicated time points (Figs 2, 4 and 6). Plating assays with phage Qβ, to verify these results, were abandoned, when *E. coli*[R1-16] supported marginal opaque plaques. Clear plaques were observed for *E. coli*[pOX38].

### R17 RNA isolation and analysis

The increase of R17 RNA during phage maturation was measured by sampling 1.5 ml culture of cells simultaneously to measuring the optical density in the liquid infection assay described above. R17 RNA was isolated using the Fermentas GENEJet RNA purification kit according to the manufacturer's recommendations and analysed by ethidium bromide gel electrophoresis. Intensity of R17 RNA was compared to the intensity of the 23S rRNA using ImageJ (ImageJ, U.S. National Institutes of Health, Bethesda, Maryland, USA, http://imagej.nih.gov/ij/, 1997–2011).

### Electron microscopy

Plate infection assays as described above were performed and agar blocks were cut with a sterile Pasteur pipette peripherally to the plaques. Samples were fixed in 2.5% glutaraldehyde (Agar Scientific, Stansted, England) in 0.1 M cacodylate buffer, pH 7.2, for 90 min at room temperature. Samples were rinsed repeatedly in 0.1 M cacodylate buffer (pH 7.2) and post-fixed with 1% osmium tetroxide (OsO_4_, Gröpl, Tulln, Austria) buffered with 0.1 M cacodylate buffer, pH 7.2, for 1 h. Subsequently, the material was rinsed twice in the buffer, dehydrated in a graded series of ethanol (including *en bloc* staining with 1% uranyl acetate in 70% ethanol for 2 h) followed by propylene oxide and embedded in Agar 100 epoxy resin (Agar Scientific, Stansted, England). Ultrathin sections (70 nm) were cut with a Reichert Ultracut S ultramicrotome and post-stained for 5 min with lead citrate before visualization with a Philips CM10 transmission electron microscope.

### Biochemical analysis

Relaxase assays on supercoiled DNA were performed as described previously (Mihajlovic *et al*., 2009). Heteroduplex substrates G2028 and IR were generated with primers G2028 fwd and G2028 rev or IR fwd and IR rev (Table S3) as described previously (Sut *et al*., 2009). T-strand cleavage and unwinding assays were performed as described (Sut *et al*., 2009).
