# Supplementary material for: An activation domain of plasmid R1 TraI protein delineates stages of gene transfer initiation
Source: Mol Microbiol. 2011 Nov 8;82(5):1071–85. doi: 10.1111/j.1365-2958.2011.07872.x (PMC3245843; doi:10.1111/j.1365-2958.2011.07872.x)
Supplement: Supplementary file 1 [file mmi0082-1071-SD1.pdf]

An activation domain of plasmid R1 TraI protein delineates stages of gene transfer initiation

Silvia Lang[1§], Paul C. Kirchberger[1§], Christian J. Gruber[1], Adam Redzej[2], Sandra Raffl[1], Guenther Zellnig[3], Klaus Zangger[2] and Ellen L. Zechner[1*]

[1]University of Graz, Institute of Molecular Biosciences, Humboldtstrasse 50, 8010 Graz, Austria; [2]University of Graz, Institute of Chemistry, Heinrichstrasse 28, 8010 Graz, Austria; [3]University of Graz, Institute of Plant Sciences, Schubertstrasse 51, 8010 Graz, Austria

key words: horizontal gene transfer, conjugation, type IV secretion, relaxase, coupling protein, bacteriophage R17

running title: Relaxase docking and transfer initiation domain

§These authors contributed equally to this study

[*]Corresponding author. Institute of Molecular Biosciences, University of Graz, Humboldtstrasse 50/1, A-8010 Graz, Austria. Tel +43 316 380 5624. Fax +43 316 380 9019. E-mail: ellen.zechner@uni-graz.at

Table S1: *E. coli* strains used in this study

Strain	Description and reference[a]
DH5α	*endA1 recA1 gyrA96 thi-l hsdR17 supE44 λ$^-$ relA1 deoR Δ(lacZYA- argF)- U169 φ80dlacZΔ(M15)* [53]
MS411	*ilvG rfb-50 thi* (M. Schembri; DTU, Denmark)
DY330	W3110 *ΔlacU169 gal 490 ts λcl857 Δ(cro-bioA)* [54]
CSH26Cm::LTL	Tc^R, CSH26 *galK::cat::loxP-Tet-loxP* [18]

[a] antibiotic resistance: Sm^R, streptomycin; Tc^R, tetracycline

Table S2: Plasmids used in this study

Conjugative plasmids	Description and reference[a]	Primer
R1	Amp^R, Cm^R, Sm^R, Km^R; IncFII, *fin*+ [55]	
R1-16	Km^R; IncFII, *fin*- [55]	
R1-16Δ*traD*	Km^R, Tc^R; IncFII, *traD*::tetRA [18]	
R1-16Δ*traI*	Km^R, Tc^R; IncFII, *traI*::tetRA [18]	
R1-16Δ*traY*	Km^R, Tc^R; IncFII, *traY*::*loxP*tetRA*loxP*; this study	traYko1_FW + traYko1_Rev
R1-16Δ*nic*	Km^R, Tc^R; IncFII, *nic*::*loxP*tetRA*loxP*; this study	oriTko1_FW + oriTko1_Rev
R1-16Δ*oriT*	Km^R, Tc^R; IncFII, *oriT*::*loxP*tetRA*loxP*; this study	oriTko1_FW + oriTko2_Rev
R1-16Δ*traM*	Km^R; IncFII; R1-16 carrying *traM* null allele; identical to R1-16M0 [37]	
pOX38	Km^R; IncFI, derivative of F [56]	
pOX38Δ*traI*	Km^R, Tc^R; IncFI; *traI*::tetRA [18]	
pOX38MK3	Km^R; IncFI; Km^R cassette inserted in the *Sal*I site of *traM* [57]	
pOX38*traD*411	Km^R; IncFI, *aph* inserted in *traD* of pOX38 [36]	

Expression vectors	Description and references[a]	Primer
CFP B	Amp^R; pBR322 expressing Cre recombinase from phage P1 [17]	
p99I+	Amp^R; pTrc99A with wild-type F *traI* [40]	
p99traI::Q369	Amp^R; p99I+ carrying 31 codon insertion at amino acid 369 [40]	
p99traI::A593	Amp^R; p99I+ carrying 31 codon insertion at amino acid 593 [40]	
p99traI::L681	Amp^R; p99I+ carrying 31 codon insertion at amino acid 681 [40]	
pAR45	pMS470Δ8 with R1 *traI* codons 310-1756 [21]	
pCG02	Cm^R; pGZ119EH [58] with R1 *traI* codons 1-992 [18]	
pHP2	Cm^R; 6.1kb *Asn*I fragment carrying R1 *traI* in pGZ119EH [34]	
pMSTraD_wt	pMS119EH [59] with wild-type R1 *traD*; this study	SS01fw + SS02rev
pMSTraD_A	pMSTraD_wt with point mutation in *traD* leading to a K198T exchange in Walker A box; this study	K198T_fw + K198T_rev
pMM_TraM	Amp^R; pMMB67EH [60] with wild-type R1 *traM*; this study	FW_TraM + Rev_TraM
pMM-M0	Amp^R; pMMB67EH with site specific R1 *traM* null mutant, R1 *oriT* and R1 *finP* [45]	
pMSYM1	Amp^R; pMS119EH with R1 *traY* insert of pGZYM1 [61]; this study	
pRelTSAFR100	Cm^R; pGZ119EH [58] with hybrid F-R1 *traI* codons 1-992; carries point mutations leading to E153D, Q193R and R210Q exchanges; this study	Set1: TraI001EcoRI_FW + PK01Rev<br>Set2: TraISeqFW12 + TraI992BamHI_Rev<br>Set3: TraI001EcoRI_FW + TraI992BamHI_Rev
pRelTSAY16FY17F	Cm^R; pGZ119EH with R1 *traI* codons 1-992; carries point mutations leading to Y16F and Y17F exchanges; this study	Set1: PK02FW + TyrtoPheRev<br>Set2: TyrtoPheFW + TraI992BamHI_Rev<br>Set3: PK02FW + TraI992BamHI_Rev
pTraIrel	Cm^R; pGZ119EH with R1 *traI* codons 1-309 [18]	

Overexpression vectors	Description and references[a]	Primer
pCG03	Km^R; pET24a (Novagen) with full-length F *traI*; carries point mutations leading to M2L, E153D, Q193R and R201Q exchanges; this study	
pet29TraI$_{1\text{-}992}$	Km^R; pET29a carrying R1 *traI* codons 1-992; this study	TraI992fw + TraI992rev
pSETraD	Amp^R; pET3a (Novagen) expressing R1 TraDΔN130 [20]	

[a] antibiotic resistance: Amp^R, ampicillin; Cm^R, chloramphenicol; Km^R, kanamycin; Sm^R, streptomycin; Tc^R, tetracycline

Table S3: Oligonucleotides used in this study

Primer for cloning	Primer sequence 5’-3’[a]
FW_TraM	GTCCC***GTCGAC***ATGGCGAAAGTGCAGGCTTATGTCA
K198T_fw	CGACGGGTACGTCAGAGGT
K198T_rev	ACCTCTGACGTACCCGTCG
oriTko1_FW	CAAAAAGGCTCAACAGGTTGGTGGTTCTCACCACCAAAAGGAGAAAAAAATCACTATAACTTCGTATAG
oriTko1_Rev	TACATTATTTAAAACATAAGTTAATGATTCAAATAGCAAATATCAACGGTGGTATATCCGGATAACTTCGTATAA
oriTko2_Rev	TTGCGTTAAATTCATTGGTGAATCATATGCGATTCACCAATGAAATCAACGGTGGTATATCCGGATAACTTCGTATAA
PK01Rev	TCCGGCCCGTCCTGTGA
PK02FW	GCAACGACTGTTTGCCCG
Rev_TraM	GATCC***CTGCAG***TTATTCCTCATCATTTTCTGGAAAG
SS01fw	GCC***GAATTC***ATGAGTTTTAACGCAAAG
SS02rev	CGTG***AAGCTT***TCAGAAATCATCTCCCG
TraI001EcoRI_FW	TT***GAATTC***ATGATGAGTATCGCGCAGG
TraI992BamHI_Rev	AA***GGATCC***TTACCCCTGTACCACCGTGAAAC
TraI992fw	ATAGTA***CATATG***ATGATGAGTATCGCGCAGGTC
TraI992rev	GCAATC***CTCGAG***CCCCTGTACCACCGTGAAAC
TraISeqFW12	TCACAGGACGGGCCGGA
traYko1_FW	AATTGAGTGAGGAGGCGTAACGCGAGAGGCGGGATAAGC**TGA**GGAGAAAAAAATCACTATAACTTCGTATAG
traYko1_Rev	CTTTGGCCCGAATAAGTCGGTTATTTGTATCTTCATCGAATATCAACGGTGGTATATCCGGATAACTTCGTATAA
TyrtoPheFW	GCCGGTAACTTTTTCACCGACAAGG
TyrtoPheRev	CCTTGTCGGTGAAAAAGTTACCGGC
**Heteroduplex primer**	**Primer sequence 5’-3’[a]**
IR fwd	CACAGCCGGATTTTGATAATGTCGCGAACACGCTGATCCCACCAACCTGTTGAGCCT
IR rev	ACATTATCAAAATCCGGCTGTGTCAGGCACTCCTTCCACAAATCGAATCATTAACTTATGTTTTAAATAATGT
G2028 fwd	CACAGCCGGATTTTGATAATGTCGCGAACACGCTGATCGGAGTGGGTTAAATTATTTACG
G2028 rev	ACATTATCAAAATCCGGCTGTGTCAGGCACTCCTTCCACAAATCACAAGTTTTTGCTGATTTGC

[a] underlined: homologous region, bold: Stop codon, bold italics: enzyme restriction site; Accession numbers: F *traI* (AP001918), R1 *traI* (AY423546), R1 *traD* (AY684127), R1 *traM* (M19710), R1 *traY* (M19710).
